# Effectiveness of Integrated Care on Delaying Progression of stage 3-4 Chronic Kidney Disease in Rural Communities of Thailand (ESCORT study): a cluster randomized controlled trial

**DOI:** 10.1186/s12882-016-0414-4

**Published:** 2017-03-02

**Authors:** Teerayuth Jiamjariyapon, Atiporn Ingsathit, Krit Pongpirul, Kotcharat Vipattawat, Suphattra Kanchanakorn, Akhathai Saetie, Duangjit Kanistanon, Patimaporn Wongprompitak, Vinai Leesmidt, Watcharapong Watcharasaksilp, Wei Wang, Anil K. Chandraker, Kriang Tungsanga

**Affiliations:** 1 Bhumirajanagarindra Kidney Institute, Phaya Thai Rd., Ratchathewi District, Bangkok, 10400 Thailand; 2 Division of Nephrology, Ramathibodi Hospital, Mahidol University, Bangkok, Thailand; 3 Department of Preventive and Social Medicine, Faculty of Medicine, Chulalongkorn University, Bangkok, Thailand; 4 Department of Immunology, Faculty of Medicine Siriraj Hospital, Mahidol University, Bangkok, Thailand; 5 Khamphaeng Phet Provincial Health Office, Ministry of Public Health, Khamphaeng Phet, Thailand; 6 Department of International Health, Johns Hopkins Bloomberg School of Public Health, Baltimore, MD USA; 7 Department of Biostatistical Science, Brigham & Women’s Hospital, Harvard Medical School, Boston, MA USA; 8 Renal Division, Brigham and Women’s Hospital, Harvard Medical School, Boston, MA USA; 9 Division of Nephrology, Faculty of Medicine, Chulalongkorn University, Bangkok, Thailand

**Keywords:** Chronic Kidney Disease, Integrated CKD care, Village health volunteers

## Abstract

**Background:**

In developing countries, renal specialists are scarce and physician-to-patient contact time is limited. While conventional hospital-based, physician-oriented approach has been the main focus of chronic kidney disease (CKD) care, a comprehensive multidisciplinary health care program (Integrated CKD Care) has been introduced as an alternate intervention to delay CKD progression in a community population. The main objective is to assess effectiveness of Integrated CKD Care in delaying CKD progression.

**Methods:**

We carried out a community-based, cluster randomized controlled trial. Four hundred forty-two stage 3-4 CKD patients were enrolled. In addition to the standard treatments provided to both groups, the patients in the intervention group also received “Integrated CKD Care”. This was delivered by a multidisciplinary team of hospital staff in conjunction with a community CKD care network (subdistrict healthcare officers and village health volunteers) to provide group counseling during each hospital visit and quarterly home visits to monitor compliance with the treatment. Duration of the study was 2 years. The primary outcome was difference of mean eGFR between the intervention and the control groups over the study period.

**Results:**

The mean difference of eGFR over time in the intervention group was significantly lower than the control group by 2.74 ml/min/1.73 m^2^ (95%CI 0.60–4.50, *p* = 0.009). Seventy composite clinical endpoints were reported during the study period with significantly different incidences between the control and the intervention groups (119.1 versus 69.4 per 1000 person-years; hazard ratio (HR) 0.59, 95% CI 0.4–0.9, *p* = 0.03).

**Conclusion:**

Integrated CKD Care can delay CKD progression in resource-limited settings.

**Trial registration:**

(NCT01978951). Prospectively registered as of December 8, 2012.

## Background

Chronic Kidney Disease (CKD) is a global public health problem, strongly associated with increased risk of death, cardiovascular events, and hospitalization [1–3]. Despite several guidelines, its prevalence is still high in developing countries [4]. A recent community-based survey in Thailand revealed that the prevalence of stage 3–5 CKD was 8.8% [5]. The total number of pre-dialysis CKD patients in Thailand has been estimated at 4.1 million, which is impossible for the currently available 450 nephrologists to deal with. This nephrologist-to-patient ratio of 1:15,000 indicated that traditional physician-based approach is inadequate for ensuring equitable access to CKD care, even under the currently successful Universal Coverage scheme. With limited national resources, delaying the progression of CKD from early to advance stages is essential for efficient utilization of the limited number of nephrologists at minimizing healthcare cost. As patients residing in rural areas are less likely to reach well-qualified personnel when compared with city dwellers [6], it is mandatory to seek other appropriate forms of renal care to delay CKD progression.

Reorienting service delivery to focus on primary care and more efficient use of healthcare professionals other than nephrologists has been one of the promising solutions. Recent evidence suggested that a comprehensive intervention by nurse practitioners supervised by a qualified nephrologist could reduce the risk of cardiovascular events among diabetic patients as well as attenuate the decline of kidney function and improve the renal outcomes in CKD patients [7–9].

Thai patients have enjoyed the unique primary health service delivery system in which health care officers at subdistrict level collaborated very well with village health volunteers (VHVs). The VHVs are villagers voluntarily recruited from their own villages to become key bridging points between subdistrict health and non-health systems. Approximately one million VHVs have covered more than 90% of all villages and therefore have become the backbone of community-based public health service in Thailand. This system has been so successful that Thailand was regarded as one of a few developing countries that have an efficient primary health care service [10–13].

Should the primary health care officers and VHVs be trained to render proper CKD care, it would be interesting to examine whether their intimate relationship and commitment to their responsible village households will result in better outcomes than the conventional physician-based care. Evidence on the effectiveness of this type of comprehensive multidisciplinary intervention in developing countries has been lacking. One reason might be that this type of complicated intervention has been developed naturally out of initiatives of different stakeholders. Our attempt to clearly define the intervention allowed for further investigation of the outcomes.

This study was aimed to compare the effectiveness on delaying CKD progression between a conventional and an integrated CKD care (ICC) provided by a multidisciplinary care team (MDCT) of hospital staffs in conjunction with the a community CKD care network (CCN, subdistrict health care officers and VHVs).

## Methods

### Study design

The ESCORT study (Effectiveness of Integrated Care on Delaying Progression stage 3–4 Chronic Kidney Disease in Rural Communities of Thailand) is a community-based, cluster randomized controlled trial. The rationale and design of this 2-year study was published elsewhere [14]. In brief, participants were stage 3-4 CKD patients, 18–70 years of age, and had diabetes and/or hypertension. They lived in the two randomized districts in Kamphang Phet province located 400 km north of Bangkok, Thailand. Of 11 districts in the province, two districts were randomly selected. Then, of the total of Stage 3-4 CKD patients in each district, 586 patients were assessed for eligibility and 442 participants were randomly selected to the study. Next, participants of each group will be further subdivided into 12 patient subgroups according to the area of their sub districts, resulting in 25–30 CKD patients per subgroup. During enrollment period, 94 cases were excluded as per exclusion criteria and 50 cases refused to participate. Those with unstable/advanced cardiovascular diseases, obstructive uropathy, HIV infection, pregnancy, body mass index (BMI) less than 18 or more than 40 kg/m^2^, untreated malignancy, urine protein-creatinine ratio exceeded 3.5 g/g creatinine, or active urinary sediments (red or white blood cells more than three and 10 cells/high power field, respectively) were excluded. The study had been conducted for 24 consecutive months (June 2011–July 2013).

### Interventions

Baseline information including past medical history, physical examination, and medications as well as blood and urine samples were collected from all subjects. While patients in both intervention and control groups received standard clinical care and medications as well as group-based educational programs during their visit to the district hospital, the intervention group also received the Integrated CKD Care program described below.

#### Integrated CKD care program

The Integrated CKD Care (ICC) program is operated by [1] a multidisciplinary care team (MDCT) of the respective district hospital, consisting of two general practitioners, two chronic care nurses, one pharmacist, one nutritionist, one physical therapist and [2] community care network (CCN) teams, each consists of one subdistrict health care officer, 3–5 village health volunteers (VHVs), and selected family members of the CKD patients residing in the respective patient subdistrict.

Instead of routine care, MDCT provided systematically comprehensive medical care and educational activities, including live demonstration of optimal diets, medication, and advice on exercise for CKD patients in each visit to the hospital. A 4-day CKD care training-course was organized for CCN members prior to study commencement. The course offered basic knowledge of CKD, NKF-K/DOQI guidelines, and optimal diet for CKD patients [15]. The content of basic medical knowledge was simplified to match with the educational level of CCN members. Dietitian [A.S.] provided essential knowledge about collection and interpretation methods of modified 24-h dietary recalls or Easy Dietary Assessment tool (EDA) [16]. In addition, each subgroup of CCN provided home visits to their respective patients at 6–8 weeks after each hospital visit. Four main assessments were done during each home visit: 24-h dietary recalls, blood pressure measurement, medication compliance monitoring including avoidance of nephrotoxic agents (e.g. non-steroidal anti-inflammatory drugs), and exercise behavior. Details of clinical intervention during hospital and home visits of both groups were reported elsewhere [14].

### Laboratory analysis

All patients were scheduled to follow-up clinical and laboratory parameters at their respective district hospitals at 3-month intervals. All blood and urine samples were analyzed with ABX Pentra 400 analyzer (HORIBA ABX S.A.S., France) located at these hospitals. All biochemistry analyses were validated according to the standard protocol of Department of Medical Sciences, Ministry of Public Health, Thailand. Serum creatinine was measured by the enzymatic method. It was standardized with standard reference material (SRM 967) by commutability study every 6 months [17]. Two out of twelve subgroups of both patient groups were randomly selected and assigned to collect 24-h urine urea nitrogen and sodium.

### Outcomes

The primary outcome of this study was the difference of mean eGFR between the two groups over the study period, measured by creatinine-based CKD-EPI equation [18]. The secondary outcomes were laboratory parameters and incidence of clinical endpoints including mortality, cardiovascular events (acute myocardial infarction and stroke), ESRD (eGFR is less than 15 ml/min/1.73 m^2^), and 50% increase in serum creatinine from baseline. In this study, acute myocardial infarction was defined as symptoms of chest pain, new ischemic pattern change of ECG and significant rising of cardiac enzymes. Acute stroke was defined as a clinically significant neurological deficit with radiographic evidence. Patients were seen at the first month and followed every 3 months afterwards for 2 years. Quality of life was assessed using the validated Thai SF-36 questionnaire [19].

### Statistical analysis

The primary outcome was analyzed based on an intention-to-treat basis. We use generalized estimating equations (GEE), adjusted for age, gender, diabetes, and hypertension, to analyze continuous variables in both primary and secondary outcomes [20]. To apply the primary outcome to real-life clinical practice, we also analyzed rate of eGFR decline based on assumption that the change was in linear pattern. The differences of rate of eGFR decline were analyzed using a linear mixed effects model, with random intercepts and slopes. The differences of incidence of clinical endpoints were analyzed using Cox-proportional-hazard models, involving survival time to the first relevant clinical endpoint in any individual patients. In this analysis, data were censored the date of last visit of patients who loss to follow-up or withdrew from the study. Descriptive statistics such as unpaired Student *t*-test and chi-square test were used to compare mean values and categorical data respectively. All statistical tests were two-tailed, using a *p*-value of less than 0.05 as being statistically significant. The data was analyzed using SPSS 16.0 (SPSS Inc., Chicago, IL, USA).

## Results

**Fig. 1:**
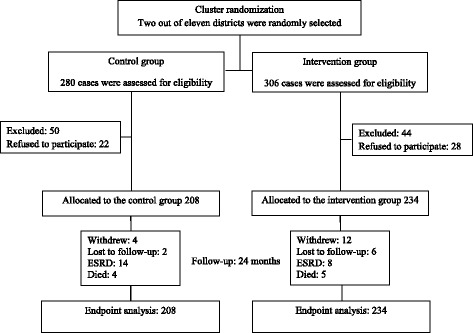
Flow chart of participants. Abbreviation: ESRD, End-stage renal disease

Findings from this study are reported according to CONSORT guidelines [21]. Four hundred and forty-two participants met the enrollment criteria, and were included into the intervention (234 cases) and control (208 cases) groups (Fig. 1). The main reasons of non-participation (94 cases) were inability to attend the proposed visits, changing point of care to another hospital outside the study region and failure to contact the patients. The study was completed in July 2013 with 24-month median follow-up time. Sixteen patients (3.6%) were lost to follow-up, eight patients (1.8%) withdrew from the study. Most of baseline clinical and laboratory characteristics, including eGFR, of the two groups were comparable; however, the levels of HbA1c, 24-h urine sodium and normalized Protein Nitrogen Appearance (nPNA) of the control group were slightly higher than intervention group (Table 1).

**Table 1:** Baseline characteristics of participants

	Control (*n* = 208)	Intervention (*n* = 234)	*P*-value
Age (years)	62.4 ± 7.9	62.3 ± 6.4	0.89
Female (%)	152 (73.1%)	170 (72.6%)	0.92
Educational status			
Elementary school or lower (%)	205 (98.6)	217 (92.7)	0.09
BMI	25.0 ± 5.5	25.7 ± 7.9	0.32
Systolic BP (mmHg)	123.8 ± 16.9	125.9 ± 15.6	0.19
Diastolic BP (mmHg)	75.6 ± 2.8	76.7 ± 2.9	0.22
Smoking (%)	7 (3.4)	4 (1.7)	0.23
Co-morbidities			
DM (%)	108 (51.9)	129 (55.1)	0.50
HT (%)	192 (92.3)	210 (89.7)	0.35
History of IHD	11 (5.2)	11 (4.7)	0.44
History of CVA	2 (0.9)	6 (2.5)	0.07
Initial laboratory parameter			
Creatinine (mg/dl)	1.53 ± 0.08	1.55 ± 0.07	0.21
eGFR (CKD-EPI, ml/min/1.73 m^2^)	41.8 ± 10.6	41.2 ± 10.3	0.32
UPCR (mg/g)	457.3 ± 700.3	442.4 ± 752.5	0.84
Hemoglobin (g/dl)	11.4 ± 2	11.6 ± 3.1	0.52
HbA1C	7.9 ± 2.4	7.3 ± 1.4	0.02
LDL (mg/dl)	113.9 ± 35.9	120.8 ± 38.5	0.06
Potassium (mEq/L)	4.6 ± 0.6	4.1 ± 0.6	0.04
Bicarbonate (mEq/L)	22.9 ± 3.7	25.4 ± 6.1	0.26
24-h u Na (mg/day)	4,485 ± 80	3,241 ± 55	0.02
24-h nPNA (g/kg/day)	1.08 ± 0.4	0.84 ± 0.2	0.01
Treatment			
No. of antihypertensive medications	2.0	2.2	0.06
ACEi/ARBs (%)	190 (91.3)	199 (85)	0.05
Statin use (%)	156 (75.5)	167 (71.4)	0.33
NSAID (%)	32 (15.4)	45 (19.2)	0.08
Aspirin (%)	133 (63.9)	85 (36.3)	0.05

Data was shown as means (standard deviation) for continuous variables and percentages for categorical variables

*Abbreviations*: *BMI* Body Mass Index, *BP* Blood Pressure, *DM* Diabetes Mellitus, *HT* Hypertension, *IHD* Ischemic Heart Disease, *CVA* Cerebrovascular accident, *eGFR* estimated Glomerular Filtration Rate, *CKD-EPI* the Chronic Kidney Disease Epidemiology Collaboration equation, *UPCR* Urine Protein-Creatinine Ratio, *HbA1C* HemoglobinA1C, *LDL* Low-density Lipoprotein, *nPNA* normalized Protein Nitrogen Appearance, *ACEi/ARBs* Angiotensin Converting Enzyme inhibitors/Angiotensin Receptor Blockers, *NSAIDs* Non-steroidal Antiinflammatory Drugs

### Effectiveness of integrated CKD care on rate of eGFR decline

**Fig. 2:**
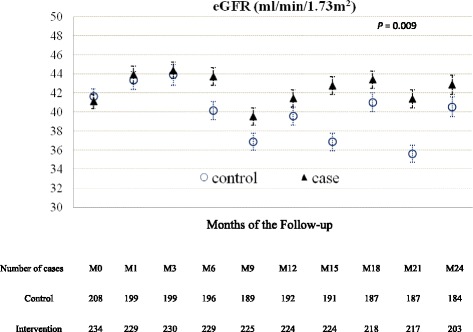
Changes in eGFR during the follow-up period (Primary outcome). GEE analyses were used to determine mean differences over time of estimated Glomerular Filtration Rate (eGFR) between intervention group and control group during the follow-up period

In adjusted analysis, the mean difference of eGFR over time in the intervention group was significantly lower than the control group 2.74 ml/min/1.13 m^2^ (95%CI 0.60–4.50, *p* = 0.009) (Fig. 2). The rate of eGFR decline in the control group and the intervention group were (−2.0) ml/min/1.73 m^2^ per year and 0.09 ml/min/1.73 m^2^ per year, respectively. The rate of eGFR decline of the intervention group was significantly lower than the control group by 2.1 ml/min/1.73 m^2^ per year (95% confidence interval (−2.8)–(−1.2), *p* = 0.001).

### Effectiveness of integrated CKD care on cardiovascular risk factors and medication use

**Fig. 3:**
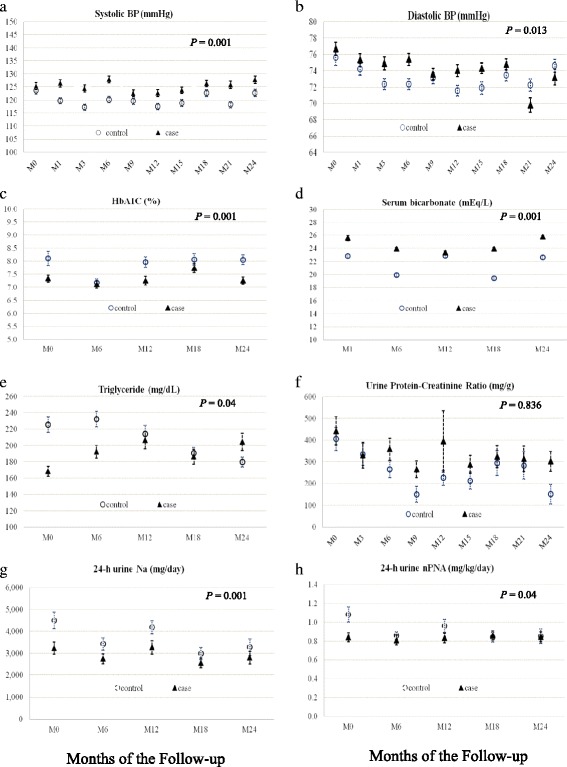
Changes in clinical and laboratory parameters during the follow-up period. GEE analyses were used to determine mean differences over time of clinical outcomes and laboratory parameters between the two groups. Change in systolic BP (**a**), diastolic BP (**b**), hemoglobin A1C (**c**), serum bicarbonate (**d**), serum triglyceride (**e**), urine protein-creatinine ratio (**f**), 24-h urine normalized protein nitrogen appearance (**g**), 24-h urine sodium (**h**) between intervention group and control group during the follow-up period

Table 2 shows the analysis of secondary outcomes, the mean values during follow-up and mean difference at the end of the study of several parameters of the intervention group were significantly lower than the control group with respect to HbA1C, serum triglycerides, 24-h urine Na, and 24-h urine nPNA. On the contrary, mean values during follow-up and mean difference of systolic and diastolic blood pressure as well as serum bicarbonate of control group were significantly lower than that of the intervention group. Urine protein-creatinine ratio and LDL-cholesterol levels of the two groups were comparable (Fig. 3).

**Table 2:** Mean levels of clinical outcomes, laboratory parameters, and medications between intervention group and control group

Variables	Mean level during follow-up		Mean difference (coefficient)	95% CI	*P*-value
	Control (*n* = 208)	Intervention (*n* = 234)			
BMI (kg/m^2^)	24.8 ± 0.2	24.9 ± 0.2	0.49	(−0.3)–1.2	0.22
eGFR (CKD-EPI, ml/min/1.73 m^2^)	39.9 ± 2.8	42.4 ± 1.5	2.74	0.7–4.8	0.009
Serum Creatinine (mg/dl)	1.6 ± 0.1	1.5 ± 0.1	−0.10	(−0.2)–(−0.02)	0.02
Systolic BP (mmHg)	120 ± 2.3	125 ± 1.9	5.37	3.4–7.3	0.01
Diastolic BP (mmHg)	73 ± 1.3	74 ± 1.8	1.23	0.3–2.2	0.01
Hemoglobin (g/dl)	11.3 ± 0.2	11.2 ± 0.3	0.45	0.36	0.42
Serum bicarbonate (mEq/L)	21.5 ± 1.7	24.5 ± 1.1	2.84	2.4–3.3	0.001
HbA1C in diabetics (%) ^a^	7.9 ± 0.4	7.3 ± 0.2	−0.57	(−0.9)–(−0.2)	0.001
LDL-C (mg/dL)	108 ± 5	107 ± 16	−1.09	(−5.6)–3.4	0.63
Triglyceride (mg/dL)	209 ± 22	192 ± 15	−18.15	(−35.5)–(−0.8)	0.04
Urine protein-creatinine ratio (mg/g)	260 ± 84	336 ± 55	11.42	(−97)–119	0.84
24-h urine Na (mg/day) ^a^	3682 ± 635	2931 ± 309	−739.0	(−1136)–(−343)	0.001
24-h urine nPNA (g/kg/day)	0.91 ± 0.1	0.84 ± 0.02	0.10	(−0.2)–(0.001)	0.049

GEE analyses were used to determine mean differences over time of clinical outcomes and laboratory parameters between the two groups

Data was shown as means (standard deviation) for continuous variables

*Abbreviations*: *GEE* generalized estimating equation

^a^Diffrences already exist at baseline

At baseline, the number of antihypertensive drugs prescribed, the percentage of patients receiving ACEI/ARBs, lipid-lowering agents, insulin, and non-steroidal anti-inflammatory drugs (NSAIDs) use were comparable between the two groups (Table 3). However, the percentage of patients in control group who received aspirin was significantly higher than the intervention group (63.9% vs. 36.3%, *p* = 0.03). At the end of the follow-up period, more patients in the intervention group received insulin than the control group (28.4% vs. 16.9%, *p* = 0.01). The percentages of medications use in the intervention group were higher than the control group but not statistically significant.

**Table 3:** Percentage of medication during the follow-up period

Medications	At baseline		*P*-value	At the end of study		*P*-value
	Control	Intervention		Control	Intervention	
	(*n* = 208)	(*n* = 234)		(*n* = 184)	(*n* = 203)	
Mean of number of antihypertensive medications	2.0 ± 0.5	2.2 ± 0.4	0.06	1.8 ± 0.4	2.7 ± 0.5	0.05
Mean of number of glucose-lowering medications	0.8 ± 0.2	1.1 ± 0.3	0.08	0.6 ± 0.1	1.7 ± 0.3	0.24
Insulin (%)	16.9	21.1	0.10	16.9	28.4	0.01
ACEi/ARBs use (%)	91.3	85	0.05	88.9	92.7	0.09
Statins use (%)	75.5	71.4	0.33	75	82.9	0.11
Antiplatelets use (%)	63.9	36.3	0.05	55.8	36.3	0.12
NSAIDs (%)	15.4	19.2	0.09	6.5	7.7	0.18

Data was shown as percent for categorical variables and compared using Chi-square test (of Fisher’s exact test)

*Abbreviation*: *ACEi/ARBs* Angiotensin Converting Enzyme inhibitors/Angiotensin Receptor Blockers, *NSAIDs* Non-steroidal Anti-inflammatory Drugs

### Effectiveness on clinical endpoints

Seventy composite clinical endpoints were reported during the study period (Table 4) with significantly different incidences between the control and the intervention groups (119.1 versus 69.4 per 1000 person-years; hazard ratio (HR) 0.59, 95% CI 0.4–0.9, *p* = 0.03). There were no significant differences in quality of life between the two groups (results not shown).

**Table 4:** Incidence of Clinical Endpoints (Cox regression analysis)^a^

Clinical outcome	Control (*n* = 208)		Intervention (*n* = 234)		*P*-value	Hazard ratio	95%CI
	No. of events	Person-years	No. of events	Person-years			
All-cause mortality	4	387.8	5	449.6	0.92	1.07	0.29–3.90
CV events^b^	4	384.0	2	448.6	0.33	0.43	0.08–2.34
ESRD^c^	14	370.5	8	439.6	0.11	0.49	0.21–1.16
50% increase in serum Cr from baseline	31	359.5	23	426.8	0.10	0.64	0.37–1.09
Composite clinical endpoints^d^	41	344.3	29	417.6	0.03	0.59	0.37–0.96

Data was analyzed by using Cox proportional-hazard model based on intention to treat basis

*Abbreviation*: *CV events* cardiovascular events

^a^In this analysis, data were censored at the date of death, the date of last visit of patients who loss to follow-up or withdrew from the study

^b^CV events in this analysis are consisted of acute myocardial infarction and stroke

^c^End-stage renal disease (ESRD) is defined as eGFR < 15 ml/min/1.73 m^2^

^d^Composite of clinical endpoints in this study is composed of CV events, ESRD, 50% increase in serum creatinine from baseline

## Discussion

This is the first, large scale, community-based randomized controlled trial evidence demonstrating that comprehensive community-based intervention by multidisciplinary care team in conjunction with a community care network teams of non-healthcare members can slow the rate of eGFR decline and is feasible and in CKD patients residing in resource-limited settings. Given similar medical care between the two groups including medication in particular ACEi/ARBs and educational materials, our study demonstrated that integrated CKD care significantly improved several clinical parameters with respect to serum bicarbonate levels, 24-h urine nPNA, 24-h urine Na, HbA1C, and serum triglyceride levels. Each parameter had been proven effectiveness on delaying CKD progression or lowering cardiovascular events by single-factorial intervention studies [22–27]. In other words, we may imply that the key factors in delaying CKD progression of Integrated CKD Care were improvement of compliance with medication and dietary control. Consequently, this intervention seems to improve some non-conventional cardiovascular risk factors and may contribute to improvement of composite clinical endpoints. We expect the results of this study will set forth a new standard of community-based CKD care for Thailand and other resource-limited countries.

According to the results of several landmark studies, the key mechanisms that could improve clinical outcomes and delay rate of eGFR decline are optimization of traditional cardiovascular risk management. Gæde P, et al. (STENO2 study) demonstrated that a long-term (mean, 7.8 years), intensified multifactorial intervention in a specialized diabetes clinic, which targeted on blood pressure <130/80 mmHg, glycosylated hemoglobin <6.5%, triglycerides <150 mg/dL, and ACEi and Aspirin use, could significantly reduce risk of cardiovascular disease (HR 0.47; 95%CI 0.24–0.73) and nephropathy (HR 0.39; 95%CI 0.17–0.87) among patients with type 2 diabetes and microalbuminuria [7]. Peeters MJ, et al. (MASTERPLAN study) proposed that significantly improved rate of eGFR decline after 2 years of follow-up was mainly related to better BP control, increased use of ACEIs/ARBs, reduction of proteinuria, and possibly increased use of active Vitamin D [8]. On the contrary, our study revealed a different perspective of CKD treatments. At the baseline, mean systolic and diastolic blood pressure of the control and the intervention groups were not statistically different. Then during the study period, despite using the same treatment guidelines and medication, mean BP overtime of the control group was significantly lower than the intervention group. However, when looking at the mean BP overtime of the two groups, mean BP overtime of the two groups, both of them were within therapeutic range. Therefore, it would not be reasonable to interprete that lowering blood pressure could be a potential reason that contributes to the delaying of eGFR decline. Even though the changes of some parameters such as blood pressure, urine protein-creatinine ratio, and LDL-cholesterol levels did not concur with findings from prior multifactorial intervention studies, these parameters were within therapeutic targets according to standard guidelines.

Interestingly, it should be noted that baseline serum bicarbonate level of the two groups was not significantly different. In this study, we found that mean bicarbonate levels over time of the control group were significantly lower than the intervention group. Therefore, it might be implied that optimization of serum bicarbonate level could lead to the delaying of rate of eGFR decline whether this was due to the intervention or not. This finding is consistent with the finding of a randomized controlled trial which Goraya N, et al. demonstrated that treatment of metabolic acidosis in patients with stage 3 chronic kidney disease with fruits and vegetables or oral bicarbonate reduces urine angiotensinogen and preserves glomerular filtration rate [25, 26].

To our knowledge, even if the mean levels over time of HbA1c, triglycerides, PNA, and urine Na of the intervention group were lower, it is still unclear whether these factors affected the rate of eGFR decline as there were baseline differences among these variables. Based on an assumption that the longitudinal data analysis (GEE) might offset the baseline differences, these better-looking secondary outcomes might be implied that intervention could improve compliance with proper dietary protein and sodium intake and medication.

Importantly, increasing CKD awareness is another possible explanation why the integrated care could affect CKD treatment outcomes. In 2009, a population-based CKD screening study in Thailand, namely Screening and Early Evaluation of Kidney Disease (SEEK) study, revealed that only 1.9% of the 3459 participants were aware that they had CKD prior to the commencement of the screening program. As our intervention was ‘tailored’ to each patient, sustainable change of patient’s awareness and behaviors that affect renal and cardiovascular conditions could be anticipated [5, 28].

The collaborative effort between MDCT and CCN was the critical component of the intervention used in this study. While MDCT provided standard medical treatment using standard clinical guidelines and essential knowledge to CKD patients using live demonstration group counseling during each hospital visit, CCN played important roles in monitoring compliance to medication, diet control, blood pressure, and exercise behavior during a series of home visits at 6–8 weeks after each hospital encounter. We also saw the benefits of detailed discussion about some clinical parameters between the patient, his or her colleagues, and the CCN team members in a cozy environment. Clinical information was conveyed in a friendly way and questions could be addressed specifically to each patient in layperson terms, under supervision of healthcare providers. This approach is critical, especially when the majority of the CKD patients are not well educated. Healthcare providers also had a better understanding of living conditions and social dynamics of the community, which could affect the progression of CKD as well as other comorbidities.

This concerted approach might help explain possible mechanisms of the renal protective effect and the composite renal end points amongst patients in the intervention group. Evidence from clinical trials conducted in developed countries revealed satisfactory cardiovascular and renal outcomes from having multidisciplinary care in hospital settings [7–9]. However, the intervention is not feasible and the findings therefore could not be generalized to developing countries where most of CKD patients lived in rural areas where specialized personnel is scarce and physician-to-patient contact time is limited [29]. While the best hospital-based CKD care is not equally available, we demonstrated that an optimal yet comprehensive set of harmonized efforts between healthcare providers and non-healthcare members is feasible and could yield similar, if not superior, clinical benefits. The implementation of the integrated CKD care model in developing countries is feasible. The use of community volunteers has been identified as one strategy to address the growing shortage of health workers, particularly in low-income countries. To our knowledge, the VHV scheme or Community Health Workers program has been established in more than 20 countries such as India, Brasil, Ethiopia, Kenya, etc. [30]. Furthermore, there is demonstrated evidence that a multifaceted intervention represents good value for money as it reduced costs but not quality of life for CKD patients (CanPREVENT study) [31].

With regard to the study which has been described above, there are some inevitable limitations. First, a cluster randomized control trial was chosen over a conventional double-blinded randomized control trial to account for the way service delivery was offered so it was possible for patients in the control group who reside in an overlapping area to be exposed to activities offered to the intervention group and vice versa. However, such phenomenon was believed to be minimal. Second, despite the randomization, there were several differences of baseline values including HbA1c, serum triglycerides, 24-h urine sodium and normalized Protein Nitrogen Appearance (nPNA), which could affect rate of eGFR decline. However, with the comparison of mean levels over time, the differences of baseline parameters may be offset. Lastly, distinguishing isolated effects of each component of our comprehensive set of interventions was difficult and therefore introduced some challenges to generalization of our findings to other settings that do not have the key CCN components.

## Conclusions

In summary, the ESCORT study, a community-based, cluster randomized controlled study of 442 stage 3-4 CKD patients with a mean follow-up duration 2 years, shows that integrated CKD care may slow the rate of eGFR decline significantly and seems to improve the number of composite clinical events.

## Abbreviations

ACEi/ARBs: Angiotensin Converting Enzyme inhibitors/Angiotensin Receptor Blockers
BMI: Body mass index
BP: Blood pressure
CCN: Community CKD care network
CKD: The Chronic Kidney Disease
CKD-EPI: The Chronic Kidney Disease Epidemiology Collaboration equation
CV events: Cardiovascular events
CVA: Cerebrovascular accident
DM: Diabetes Mellitus
eGFR: estimated Glomerular Filtration Rate
GEE: Generalized estimating equation
HbA1C: HemoglobinA1C
HT: Hypertension
ICC: Integrated CKD care
IHD: Ischemic Heart Disease
LDL: Low-density Lipoprotein
MDCT: Multidisciplinary care team
nPNA: normalized Protein Nitrogen Appearance
NSAIDs: Non-steroidal anti-inflammatory drugs
SRM: Standard reference material
UPCR: Urine protein-creatinine ratio
VHVs: Village health volunteers
